# Decreased Frequencies of Circulating Follicular Helper T Cell Counterparts and Plasmablasts in Ankylosing Spondylitis Patients Naïve for TNF Blockers

**DOI:** 10.1371/journal.pone.0107086

**Published:** 2014-09-09

**Authors:** María-Belén Bautista-Caro, Irene Arroyo-Villa, Concepción Castillo-Gallego, Eugenio de Miguel, Diana Peiteado, Chamaida Plasencia-Rodríguez, Alejandro Villalba, Paloma Sánchez-Mateos, Amaya Puig-Kröger, Emilio Martín-Mola, María-Eugenia Miranda-Carús

**Affiliations:** 1 Department of Rheumatology, Hospital Universitario La Paz-IdiPAZ, Madrid, Spain; 2 Laboratorio de Inmuno-Oncología, Hospital General Universitario Gregorio Marañón, Madrid, Spain; Institute of Immunology, Rikshospitalet, Norway

## Abstract

Follicular helper T cells (Tfh), localized in lymphoid organs, promote B cell differentiation and function. Circulating CD4 T cells expressing CXCR5, ICOS and/or PD-1 are counterparts of Tfh. Three subpopulations of circulating CD4+CXCR5+ cells have been described: CXCR3+CCR6- (Tfh-Th1), CXCR3-CCR6+ (Tfh-Th17), and CXCR3-CCR6- (Tfh-Th2). Only Tfh-Th17 and Tfh-Th2 function as B cell helpers. Our objective was to study the frequencies of circulating Tfh (cTfh), cTfh subsets and plasmablasts (CD19+CD20-CD27+CD38^high^ cells), and the function of cTfh cells, in patients with Ankylosing Spondylitis (AS). To this end, peripheral blood was drawn from healthy controls (HC) (n = 50), AS patients naïve for TNF blockers (AS/nb) (n = 25) and AS patients treated with TNF blockers (AS/b) (n = 25). The frequencies of cTfh and plasmablasts were determined by flow cytometry. Cocultures of magnetically sorted CD4+CXCR5+ T cells with autologous CD19+CD27- naïve B cells were established from 3 AS/nb patients and 3 HC, and concentrations of IgG, A and M were measured in supernatants. We obseved that AS/nb but not AS/b patients, demonstrated decreased frequencies of circulating CD4+CXCR5+ICOS+PD-1+ cells and plasmablasts, together with a decreased (Tfh-Th17+Tfh-Th2)/Tfh-Th1 ratio. The amounts of IgG and IgA produced in cocultures of CD4+CXCR5+ T cells with CD19+CD27- B cells of AS/nb patients were significantly lower than observed in cocultures established from HC. In summary, AS/nb but not AS/b patients, demonstrate a decreased frequency of cTfh and plasmablasts, and an underrepresentation of cTfh subsets bearing a B helper phenotype. In addition, peripheral blood CD4+CXCR5+ T cells of AS/nb patients showed a decreased capacity to help B cells ex vivo.

## Introduction

Ankylosing spondylitis (AS) is the prototype of Spondyloarthritis (SpA) [1], [2], a group of diseases sharing clinical, radiographic and genetic features [1], [2]. Despite intensive research, the pathogenesis of SpA is not well understood, and evidence suggesting the implication of either autoinflammatory or autoimmune mechanisms has been reported [3].

The role of B cells and of humoral immunity in SpA is not clear. Several autoantibodies have been observed in patients with SpA [4]–[7], but their poor sensitivity or specificity have not allowed to establish a clear pathogenic association. More recently, reactivity against CD74 has been proposed as a marker for SpA [8], [9]. Also, increased numbers of CD5+ B lymphocytes have been described in SpA [10]. Furthermore, it has been reported that some patients with AS seem to benefit from B cell depleting therapeutic strategies [11].

Follicular helper T cells (Tfh) are a major subset of effector T lymphocytes specialized in the provision of help to B cells [12]–[17], and characterized by their surface phenotype (CD4+CXCR5+ICOS+PD-1+), cytokine profile (IL-21, IL-10, IL-17) and transcriptional program (BCL-6) [12]–[17]. Tfh cells seem to be implicated in autoimmunity [18], and increased numbers are found in murine models of Systemic Lupus Erythematosus (SLE) [18]–[20] and inflammatory arthritis [21]; furthermore, strategies directed at reducing Tfh cell generation ameliorate disease manifestations in these animal models [20], [22].

The initial definition of Tfh cells was based on both their phenotype and their characteristic location in secondary lymphoid organs [12]–[17]. Several reports have subsequently described circulating populations of CD4 T cells that express CXCR5 and share both phenotypical and functional properties of classical Tfh cells [23]–[26]. Increased frequencies of circulating Tfh cell counterparts (cTfh), have been associated with autoimmune diseases such as SLE [23], Rheumatoid Arthritis (RA) [27], Sjögren's Syndrome [28], autoimmune thyroiditis [29], chronic active hepatitis [30] and myasthenia gravis [31]. More recently, three subpopulations of cTfh cells have been described, based on their differential expression of the chemokine receptors CXCR3 and CCR6 and on their distinct functional capacities [24]. An altered balance of these cTfh subsets has been associated with autoimmunity in juvenile dermatomyositis and SLE [24], [32].

The features of cTfh cells or their subsets in SpA have not been fully characterized, and to our knowledge only two articles with discordant results have been published on this matter [33], [34]. Therefore, our objective was to study the frequency and function of cTfh cells, together with the frequency of cTfh subsets and plasmablasts (CD19+CD20-CD27+CD38^high^ B cells), in patients with Ankylosing Spondylitis (AS). We observed that AS patients naïve for TNF blockers (AS/nb) but not those receiving TNF blocking agents (AS/b), demonstrate a decreased frequency of cTfh and plasmablasts, and an underrepresentation of cTfh subsets bearing a B helper phenotype. In addition, peripheral blood CD4+CXCR5+ T cells of AS/nb patients showed a decreased capacity to help B cells ex vivo.

## Patients and Methods

### Ethics Statement

The study was approved by the Hospital La Paz - IdiPAZ Ethics Committee, and all subjects provided written informed consent according to the Declaration of Helsinki.

### Patients

Peripheral blood was obtained from 25 AS patients who had never received TNF blockers (AS/nb), 25 AS patients treated with TNF blockers (AS/b) and from 50 age and gender-matched healthy controls (HC). AS was diagnosed according to the 1984 modified New York criteria [35]. For patients receiving TNF blockers, blood was drawn immediately before the infusion/administration of the drug. All subjects were of Western European descent.

Among AS/nb patients, 16 were taking non-steroidal anti-inflammatory drugs (NSAIDs) and 4 were receiving sulfasalazine (SSZ); 5 of them did not take any medication regularly. Among AS/b patients, 20 were receiving infliximab, 4 etanercept and 1 adalimumab. In addition to TNF blockers, 7 patients were taking NSAIDs, and 8 were taking SSZ. Clinical characteristics of all patients are shown in Tables 1 and 2.

**Table 1:** Clinical characteristics of AS/nb and AS/b patients.

	AS/nb (n = 25)	AS/b (n = 25)
Age (years); median (IQR)	56 (45.5–65.5)	53 (45–61.5)
Male; n° (%)	12 (48)	16 (64)
HLA-B27+; n° (%)	22 (88)	23 (92)
Duration of symptoms (yrs); median (IQR)	25.5 (17.5–35)	24.5 (14–36)
Time since diagnosis (yrs); median (IQR)	11.5 (5.5–20)	15 (9–22.5)
Time on TNF blockers (yrs); median (IQR)	-	7 (5–9)
BASDAI; median (IQR)	3.9 (2.8–4.8)	2.6 (1.3–6.1)
BASFI; median (IQR)	1.8 (0.5–4.9)	3 (0.6–5.8)
ASDAS-ESR; median (IQR)	2.14 (1.89–2.72)	2.22 (1.41–3.15)
ASDAS-CRP; median (IQR)	2.55 (1.9–3.13)	2.34 (1.07–3.02)
CRP; median (IQR)	7.37 (2.51–11.1)	4.52 (1.44–15.4)
Pt. global assessment; median (IQR)	3 (2–6)	3 (2–6.5)

AS/nb: AS patients naïve for TNF blockers; AS/b: AS patients treated with TNF blockers.

**Table 2:** Number of patients according to disease activity state based on ASDAS-CRP values [38].

ASDAS-CRP = = >	< 1.3 (inactive disease)	1.3–2.1 (moderate activity)	2.1–3.5 (high activity)	>3.5 (very high activity)
*AS/nb (# of patients)*	2	7	13	3
*AS/b (# of patients)*	7	1	13	4

### Isolation of CD4+ T cells and B cells from human peripheral blood

Peripheral blood mononuclear cells (PBMCs) were separated immediately after blood sample collection, by Ficoll-Hypaque (GE Healthcare Biosciences AB, Uppsala, Sweden) density gradient centrifugation. CD4+ T or B cells were purifed from freshly isolated PBMCs by exhaustive immunomagnetic negative selection in an Automacs (Miltenyi Biotec, Bergisch Gladbach, Germany), using the “CD4+ T Cell Isolation Kit” or the “B Cell Isolation Kit II” from Miltenyi Biotec. Isolated CD4+ T cells or CD19+ B cells were >98% pure. CXCR5+ and CXCR5- subpopulations were subsequently isolated from total CD4+ T cells using PE-labeled CXCR5 microbeads (Miltenyi Biotec). Naïve (CD19+CD27-) and memory (CD19+CD27+) B cells were selected from total CD19+ B cells using CD27+ microbeads (Miltenyi Biotec). T and B cell subpopulations were >98% pure and used immediately after isolation.

### B cell/T cell cocultures

To assess the functional capacity of circulating CD4+CXCR5+ T cells, sorted CXCR5+ cells (2×10^5^ cells/well) were cocultured for 13 days with autologous naïve B (CD19+CD27-) cells (1×10^5^ cells/well) in U-bottom 96-well plates containing RPMI 1640 medium (Lonza, Alendale, NJ, USA) with 10% FCS, 2 mM L-glutamine, 50 U/ml penicillin, 50 µg/ml streptomycin and 50 µM 2-mercapto-ethanol. Endotoxin-reduced staphylococcal enterotoxin B (SEB) (1 mg/ml) (Sigma-Aldrich) was also added to the cultures, since the production of immunoglobulins in Tfh/B cell cocultures has been shown to depend on cognate T/B cell interactions [24]. For comparison, autologous cocultures of CD4+CXCR5+ T cells with CD19+CD27+ memory B cells and cocultures of CD4+CXCR5- T cells with naïve or memory B cells were also established. Concentrations of IgG, IgA and IgM were measured in coculture supernatants at different time points by ELISA.

### Cell Surface Staining and Flow Cytometry

The frequency and phenotype of Tfh-like cells and of plasmablasts present in the peripheral blood of AS patients and HC was assessed by flow cytometry after staining freshly isolated PBMCs with antibodies directed against surface phenotypical markers. Fluorochrome-conjugated mAbs from BD Pharmingen (San Diego, CA, USA) were used to examine the expression of CD3, CD4, CD8, CXCR5, ICOS, PD-1, CCR6, CXCR3, CD19, CD20, CD27 and CD38 in a FACSCalibur flow cytometer with CellQuest software (BD Biosciences).

### ELISAs

Cell-free coculture supernatants were collected and stored at −80°C. The concentrations of immunoglobulins were measured by ELISA. In brief, 96-well plates (MaxiSorp, Thermo Fisher Scientific, Waltham, MA, USA) were coated overnight at 4°C with 10 µg/ml mouse monoclonal anti-human IgG, IgA or IgM (AbD Serotec, Munich, Germany), and subseqently blocked with 2% BSA/PBS. Standard curves of human IgG, IgA or IgM (Sigma-Aldrich) together with culture supernatants diluted in 2% BSA/PBS were incubated for 3 hours at room temperature, washed and developed with HRP-conjugated goat anti-human IgG, IgA or IgM (ABD serotec) followed by TMB substrate solution (BD-Pharmingen). Absorbance was measured at 450 nm in a Synergy H4 Hybrid Multi-Mode Microplate Reader (BioTec Instruments, Inc., Winoosi, VT, USA).

### Determination of serum IgG, IgA and IgM concentrations

Sera from all studied subjects were collected on the day when phenotypical studies were done, and stored at −80°C. Concentrations of IgG, IgA and IgM were subsequently examined in sera of AS/nb, AS/b patients and HC by nephelometry in an Immage 800 Immunochemistry System (Beckman Coulter, Brea, CA, U.S.A). For AS/b patients, determinations of Igs were also performed in serum that had been collected just before initiation of treatment with TNF blockers.

### Statistical Analysis

Comparison between groups was by Mann-Whitney or Kruskal-Wallis test. When appropriate, Bonferroni correction for multiple comparisons was applied. Correlations were analyzed using Spearman's rank correlation coefficients. All analyses were performed using Prism version 5.0 software (GraphPad Software, San Diego, CA, USA).

## Results

### Patients with AS naïve for TNF blockers demonstrate decreased numbers of circulating Tfh counterparts

**Figure 1:**
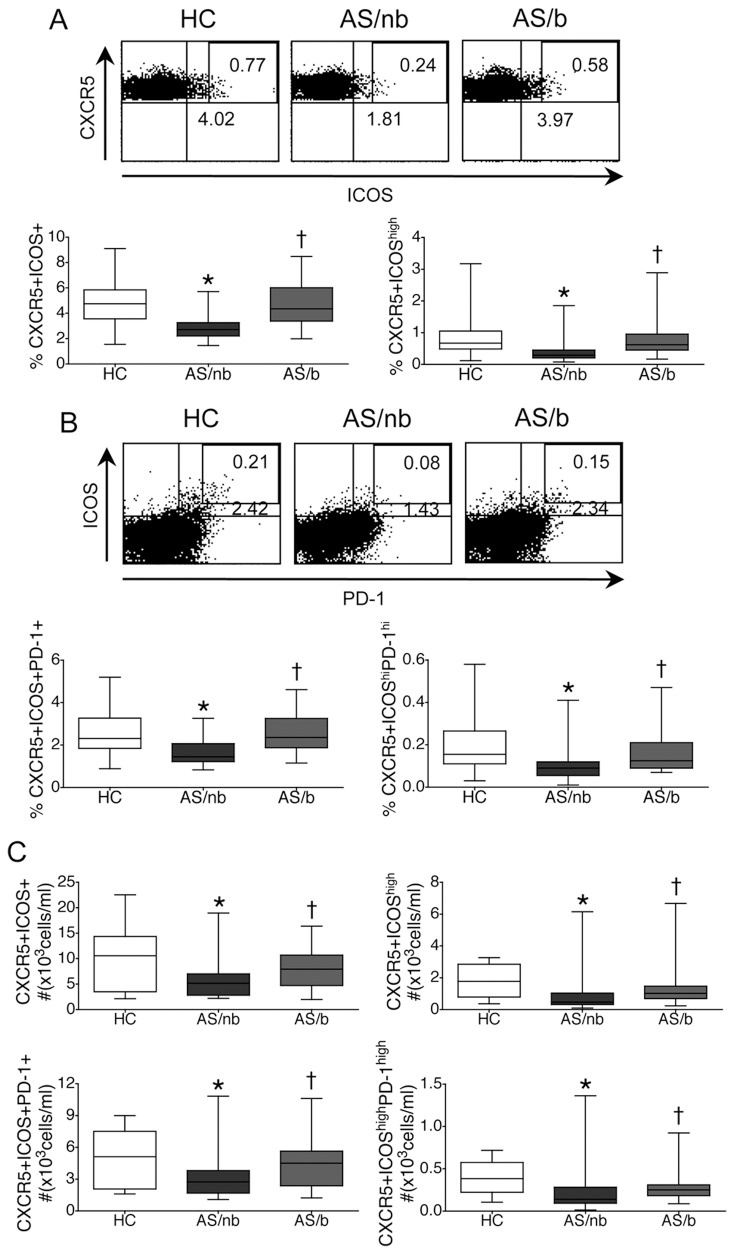
Numbers of circulating Tfh counterparts (cTfh) in patients with AS. AS/nb but not AS/b patients demonstrate decreased frequencies and absolute numbers of cTfh. A, B. Frequency of cTfh in HC, AS/nb and AS/b patients. Representative dot plots demonstrate ICOS and CXCR5 expression (A) or ICOS and PD-1 expression (B) in cells gated for CD3, CD4 and CXCR5. C. Absolute numbers of circulating Tfh counterparts (cTfh) in HC, AS/nb and AS/b patients. Box and whiskers plots represent the median, interquartile range, maximum and minimum values calculated from 25 AS/nb patients, 25 AS/b patients and 50 HC. *p<0.0001 vs HC; † p<0.01 vs AS/nb patients.

We first sought to examine the expression of Tfh phenotypical surface markers on peripheral blood CD4+ T cells. The frequency of CXCR5+ cells contained in circulating CD4+ T lymphocytes was not different among the three groups of studied subjects: 18.3±5.8 % in HC, 18.6±7.6 % in AS patients naïve for TNF blockers (AS/nb) and 18.1±5.6 % in AS patients treated with TNF blockers (AS/b) (mean ± SD). However, the frequencies of circulating total CD4+CXCR5+ICOS+ and of CD4+CXCR5+ICOS^high^, together with the frequencies of total CD4+CXCR5+ICOS+ PD-1+ and of CD4+CXCR5+ICOS^high^PD-1^high^ T cells, that are currently considered as circulating counterparts of classical Tfh cells (cTfh) [23], [25], [26], were significantly decreased in AS/nb patients (Figure 1, A, B) but were not different from controls in AS/b patients (Figure 1, A, B). In parallel, the absolute numbers of cTfh were also decreased in AS/nb patients (Figure 1 C).

### Patients with AS naïve for TNF blockers demonstrate an altered balance of circulating Tfh subsets

**Figure 2:**
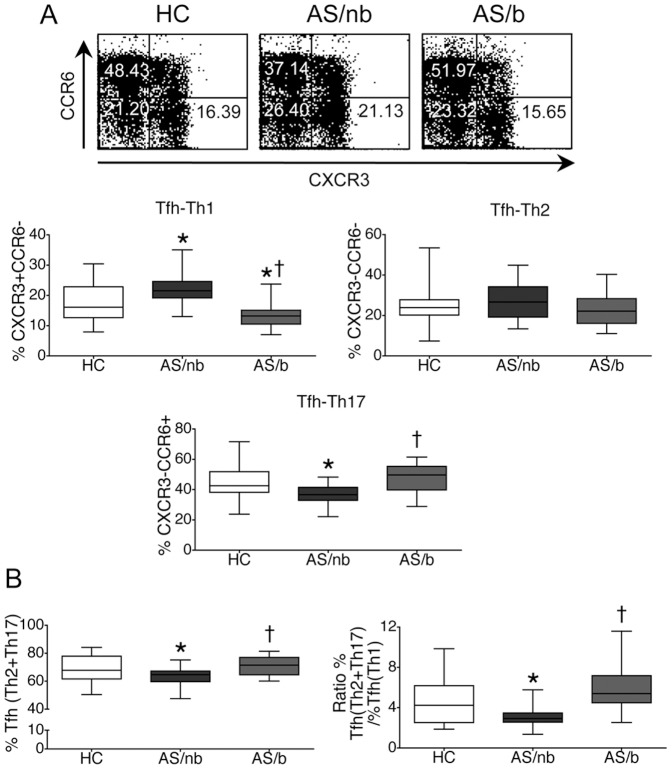
Frequency of circulating Tfh subsets in patients with AS. A. AS/nb, but not AS/b patients, demonstrate an increased frequency of circulating CD4+CXCR5+CXCR3+CCR6- (Tfh-Th1) cells and a decreased frequency of CD4+CXCR5+CXCR3-CCR6+ (Tfh-Th17) cells as compared with HC, whereas the frequency of CD4+CXCR5+CXCR3-CCR6- (Tfh-Th2) cells is not different among the three groups. Representative dot plots demonstrate CXCR3 and CCR6 expression in cells gated for CD3, CD4 and CXCR5. B. Underrepresentation of cTfh subsets with a B cell helper phenotype (Tfh-Th17 plus Tfh-Th2 cells) in AS/nb but not in AS/b patients. Box and whiskers plots represent the median, interquartile range, maximum and minimum values calculated from 25 AS/nb patients, 25 AS/b patients and 50 HC. *p<0.05 vs HC, ^†^p<0.005 vs AS/nb.

We then examined the frequency of cTfh cell subsets as described by Morita et al. [24], based on the combined expression on CD4+ T cells of CXCR5 with CCR6 or CXCR3, that are characteristic chemokine receptors expressed on Th17 or Th1 cells, respectively. AS/nb patients demonstrated an increased frequency of circulating CD4+CXCR5+CXCR3+CCR6- (Tfh-Th1) cells together with a decreased frequency of CD4+CXCR5+CXCR3-CCR6+ (Tfh-Th17) cells, whereas the frequency of CD4+CXCR5+CXCR3-CCR6- (Tfh-Th2) cells was not different from controls (Figure 2A). Furthermore, the sum of %Tfh-Th2 plus %Tfh-Th17 cells and the ratio (%Tfh-Th2+%Tfh-Th17)/%Tfh-Th1, were decreased in AS/nb patients (Figure 2B). That is, AS/nb but not AS/b patients demonstrated a relative deficiency of cTfh cell subsets bearing a phenotype associated with B cell helping capacity [24].

### Patients with AS naïve for TNF blockers demonstrate decreased numbers of circulating plasmablasts

**Figure 3:**
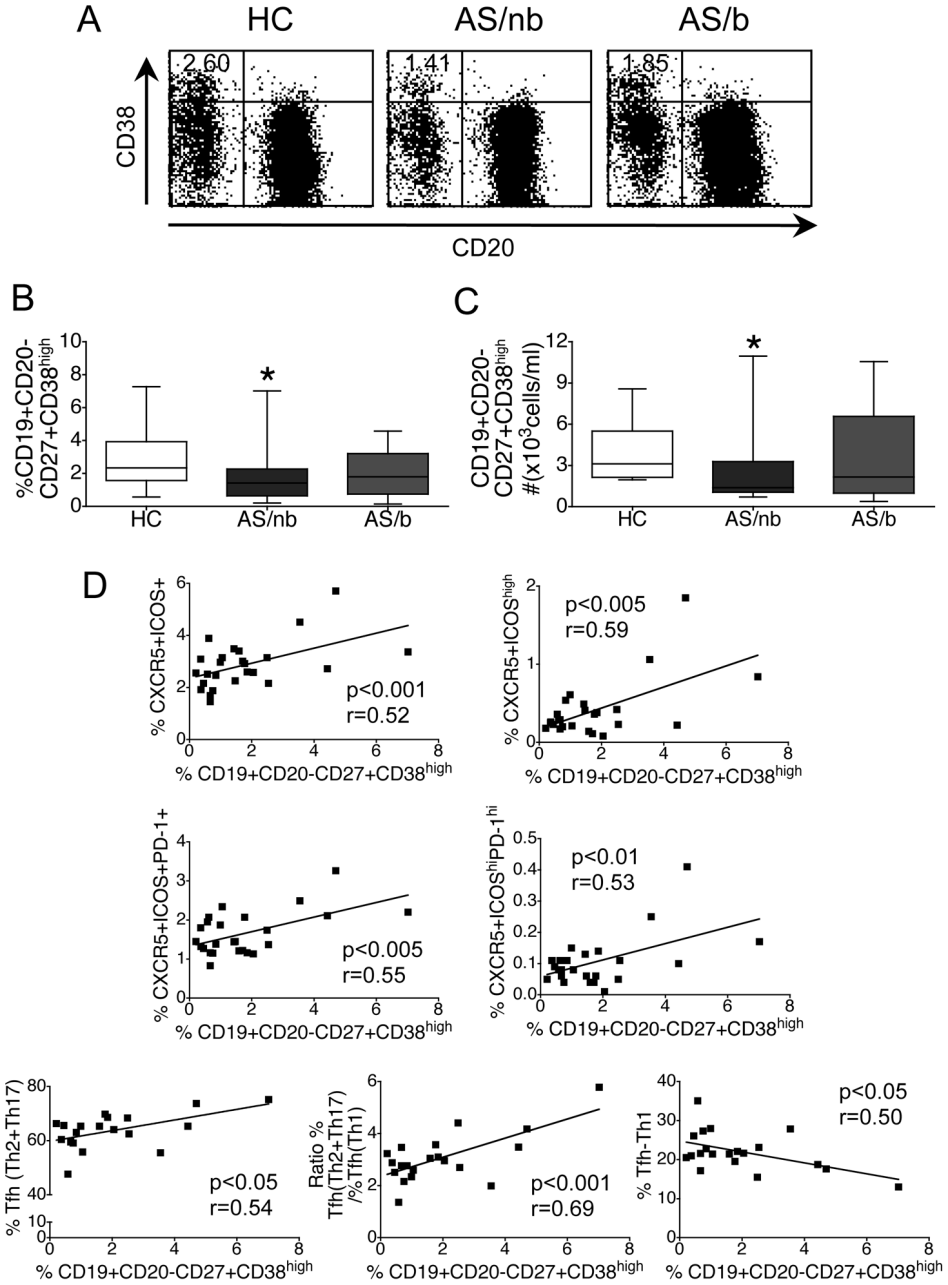
Numbers of circulating plasmablasts in patients with AS. A, B. AS/nb but not AS/b patients demonstrate a decreased frequency of circulating plasmablasts. Shown are representative dot plots of CD20 and CD38 expression on CD19+ cells (A). C. Decreased absolute numbers of circulating plasmablasts in AS/nb patients. B and C represent box and whiskers plots from 25 AS/nb patients, 25 AS/b patients and 50 HC; * p <0.005 vs HC. D. The frequency of circulating plasmablasts in AS/nb patients is positively correlated with the frequency of circulating Tfh counterparts, with the frequency of Tfh(Th2+Th17) and with the ratio [%Tfh(Th2+Th17)]/%Tfh(Th1) cells, and negatively correlated with the frequency of Tfh-Th1 cells.

The frequency of circulating plasmablasts among CD19+ cells, defined as CD19+CD20-CD27+CD38^high^ B cells, was decreased in AS/nb but not in AS/b patients (Figure 3, A, B). In parallel, the absolute number of circulating plasmablasts was also decreased in AS/nb (Figure 3C).

Interestingly, in AS/nb patients, the frequency of circulating plasmablasts was positively correlated not only with the frequency of cTfh counterparts, but also with the sum %Tfh-Th2+%Tfh-Th17 and with the ratio (%Tfh-Th2+%Tfh-Th17)/%Tfh-Th1 cells (Figure 3D). Conversely, the frequency of circulating plasmablasts was negatively correlated with the frequency of Tfh-Th1 cells in AS/nb (Figure 3D).

In addition, the absolute number of circulating plasmablasts was positively correlated with the absolute numbers of CD4+CXCR5+ICOS+ (r = 0.40, p<0.05), CD4+CXCR5+ICOS^high^ (r = 0.46, p<0.05) and CD4+CXCR5+ICOS^high^PD-1^high^ T cells (r = 0.41, p<0.05).

### Functional capacity of circulating CD4+CXCR5+ T cells

We then went on to examine the functional capacity of circulating total CD4+CXCR5+ T cells, which in AS/nb patients contain decreased proportions of cTfh, together with decreased proportions of cTfh cell subsets bearing a phenotype associated with B cell helping capacity. To this end, cocultures of sorted CD4+CXCR5+ T cells from 3 AS/nb patients and 3 age and gender-matched healthy controls were established with autologous naïve CD19+CD27- B cells; secretion of IgG and IgA in supernatants were measured as a readout of B cell maturation. All three patients were male, aged 45, 51 and 57 years and were taking NSAIDs. Two of them were HLA B27+ and one was HLA B27-. Duration of symptoms was 28, 33 and 36 years and time since diagnosis was 25, 27 and 13 years, respectively. Their ASDAS-CRPs were 3.4, 2.75 and 2.05.

**Figure 4:**
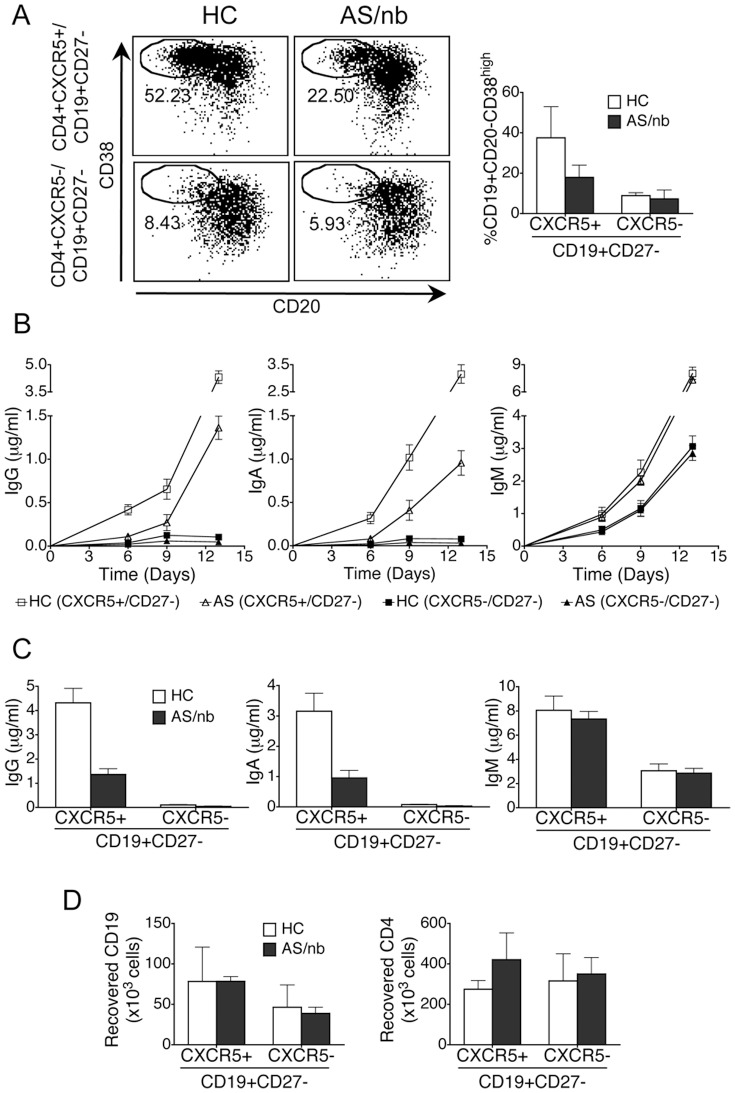
Functional capacity of circulating CD4+CXCR5+ T cells. CD4+CXCR5+ T cells isolated from peripheral blood of HC are more efficient than cells from AS/nb patients at inducing maturation of cocultured autologous naïve B cells. A. CD19+CD20-CD38^high^ plasmablasts in 9-day cocultures of naïve B cells from AS/nb patients or HC with autologous CXCR5+ or CXCR5- CD4+ T cells. B, C. Concentrations of IgG, A and M at different time points (B) or at 13 days (C) in cocultures of naïve B cells from AS/nb patients or HC with autologous CXCR5+ or CXCR5- CD4+ T cells. D. Recovered viable B and T cells in 9-day cocultures of naïve B cells from AS/nb patients or HC with autologous CXCR5+ or CXCR5- CD4+ T cells. Line and bar graphs represent the mean and SD of 3 independent experiments.

In cocultures of naïve B cells with CD4+CXCR5+ T cells, increased proportions of CD19+CD20-CD38^high^ plasmablasts (Figure 4A) were observed; in addition, IgG and IgA were detected in supernatants from the 6th day on, with increasing concentrations up to the 13th day (Figure 4B,C). Interestingly, in cocultures established with T and B cells of AS/nb patients, lower proportions of CD19+CD20-CD38^high^ plasmablasts and lower concentrations of both IgG and IgA were observed as compared with with HC (Figure 4, A-C). In contrast, the amount of secreted IgM was not different in cocultures of naïve B cells with CD4+CXCR5+ T cells established from HC or AS/nb patients (Figure 4B, C). Of note, the number of recovered viable B cells was not different in cocultures established from HC or AS/nb patients (Figure 4D).

No IgG or IgA could be detected in coculture supernatants of naïve B cells with CD4+ CXCR5- T cells from either AS/nb patients or healthy controls (Figure 4B,C), which only produced low amounts of IgM (Figure 4B, C). This was not attributable to poor T cell survival since the amount of recovered viable T cells was comparable in cocultures of naïve B cells established with either CXCR5+ or CXCR5- CD4+ T cells (Figure 4D).

In addition, isolated CD4+CXCR5+ of HC or AS/nb patients were more efficient than CD4+CXCR5- T cells at helping memory CD19+CD27+ B cells to secrete IgG, IgA and IgM. No differences between HC and AS/nb were observed when measuring the secreted amounts of IgG, IgA or IgM in these CD4+CXCR5+/CD19+CD27+ or CD4+CXCR5-/CD19+CD27+ cocultures (data not shown).

### Serum levels of IgG, IgA and IgM in AS/nb and AS/b patients, and their relation with disease activity and cTfh numbers

**Figure 5:**
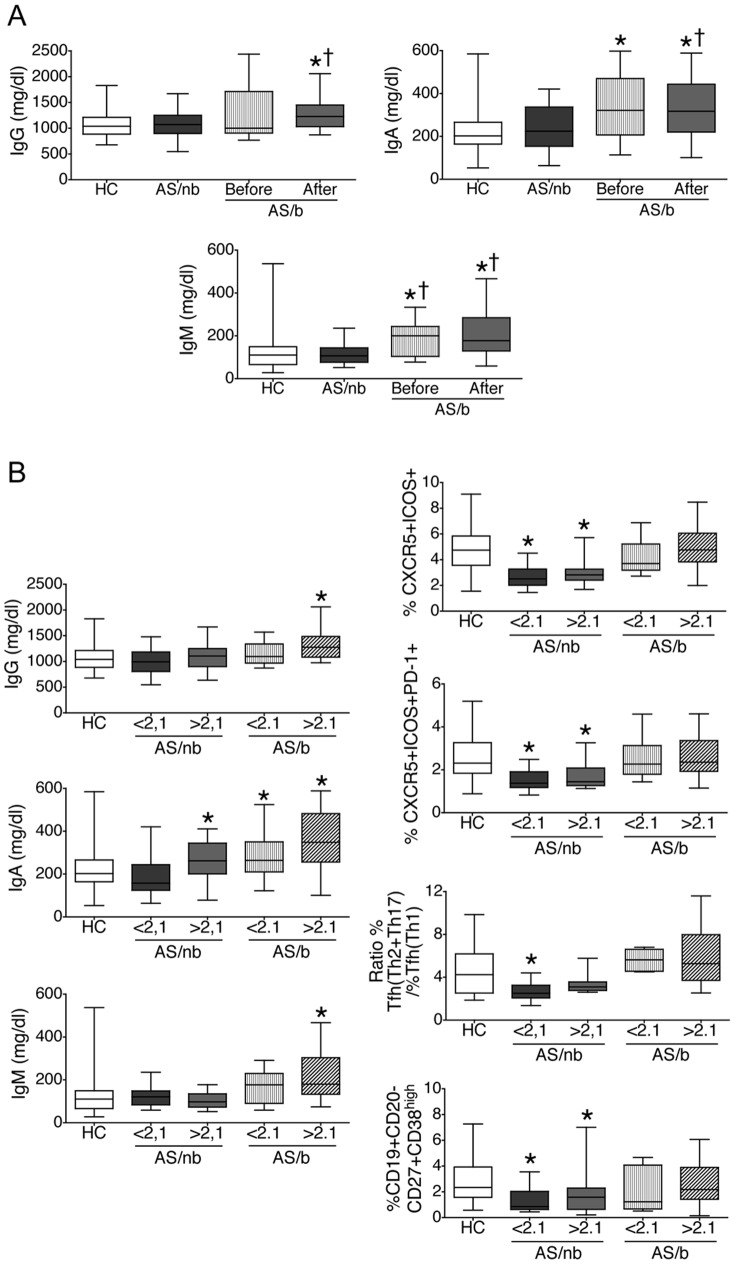
Serum levels of IgG, IgA and IgM in patients with AS. A. IgG, IgA and IgM were determined by nephelometry in the serum of AS/nb and AS/b patients. For AS/b patients, determinations were not only done in sera collected on the day when phenotypical studies were done, but also on sera that had been taken just before initiation of treatment with TNF blockers. B. Relation of serum Ig concentrations with cTfh, circulating plasmablasts and disease activity. Shown are cTfh proportions, cTfh subset ratio and circulating plasmablast proportions together with serum IgG, IgA and IgM concentrations in HC, AS patients with inactive disease or moderate disease activity (ASDAS-CRP < 2.1) [38] and AS patients with high or very high disease activity (ASDAS-CRP > 2.1) [38]. Normal or elevated serum Ig levels are observed despite the presence of decreased or normal cTfh and plasmablasts, respectively. Note that serum Ig concentrations vary with disease activity whereas cTfh and plasmablast numbers do not. Box and whiskers plots represent the median, interquartile range, maximum and minimum values. *p<0.05 vs HC, ^†^p<0.05 vs AS/nb.

As previously described [36], [37], serum IgA levels were elevated in AS patients (Figure 5A, B). Interestingly, among our AS/nb patients, serum IgA was ony increased in those who had high or very high activity as determined by an ASDAS-CRP > 2.1 [38] (Figure 5B). In contrast, all of our AS/b patients demonstrated elevated serum IgA, even those with inactive disease or moderate activity, as determined by an ASDAS-CRP < 2.1 [38], although levels were higher in patients with high or very high activity (Figure 5B). In addition, AS/b patients had elevated serum IgG and IgM levels, that were more marked in subjects with high or very high activity (Figure 5B). Importantly, increased serum IgA and IgM levels in this group were already present in sera taken just before initiation of treatment with TNF blockers (Figure 5A). This is consistent with previous observations indicating that AS patients with severe disease can demonstrate increased levels of all three IgG, IgA and IgM [39]. Interestingly, the above described normal or increased Ig levels did not parallel the decreased or normal cTfh and plasmablast numbers observed in AS/nb or AS/b subjects, respectively (Figure 5B). That is, AS/nb patients showed normal or increased IgA levels in the presence of decreased cTfh and plasmablasts and altered cTfh subset ratio (Figure 5B). In addition, AS/b patients demonstrated increased Ig levels in the presence of cTfh, cTfh subset ratio and plasmablast values that were not different from HC (Figure 5B). Furthermore, whereas serum Ig concentrations paralleled disease activity, cTfh and plasmablast numbers did not (Figure 5B).

## Discussion

Spondyloarthritis (SpA) and their prototype disease, AS, are typically not associated with circulating autoantibodies [1]–[3] and therefore, the role of B cells or B-helper T cells (Tfh) in their pathogenesis has not been thoroughly investigated. There is more recent evidence indicating that B cells may indeed be implicated in SpA [8]–[11], and we deemed it interesting to study the frequency of cTfh cells and plasmablasts in AS.

We have herein described that AS/nb patients demonstrate a decreased frequency of cTfh cells, together with a decreased frequency of circulating plasmablasts. Furthermore, an altered proportion of cTfh subpopulations is observed: AS/nb patients showed a predominance of Tfh-Th1 cells, that lack B cell helping capacity in healthy subjects [24], over Tfh-Th17 and Tfh-Th2 cells, that do have B cell helping capacity as described by Morita et al. [24]. In contrast, AS/b patients do not display these alterations. Discordance with previously published observations [33], [34] may be attributable to different treatment, ethnicity, disease duration or activity.

Because AS/nb patients were not receiving methotrexate or other drugs that may interfere with the cell cycle, the observed alterations in cTfh and plasmablasts in this patient group are not attributable to a drug effect. Conversely, the finding of normal frequencies of cTfh and plasmablasts in AS patients receiving TNF blockers suggests that these drugs may restore the initial immune alteration. Because the ASDAS-CRP and ASDAS-ESR indexes were comparable beween AS/nb and AS/b patients, the observed differences in cTfh numbers and cTfh subset balance do not seem related with different disease activity. In addition, there was no correlation of cTfh cell numbers or subsets with disease activity in either group; this parallels the observation that increased numbers of cTfh in SLE do not associate with disease activity [23] but are related to the pathogenesis of the disease [23].

Of note, the ex vivo functional studies herein described demonstrated that isolated peripheral blood CD4+CXCR5+ T cells are indeed able to provide B cell help as previously reported [24]–[26]: CD4+CXCR5+ but not CD4+CXCR5- T cells, induced IgG and IgA secretion in cocultured CD19+CD27- B cells. In the presence of CD4+CXCR5- T cells, CD19+CD27- B cells only produced low amounts of IgM as expected [24], [25]. In addition, CD4+CXCR5+ T cells were more efficient than CD4+CXCR5- T cells at helping memory CD19+CD27+ B cells to produce Igs. Interestingly, we observed that total CD4+CXCR5+ T cells from AS/nb patients, containing decreased proportions of cTfh and an altered balance of cTfh subpopulations, do have a reduced capacity to promote maturation of naïve B cells, as indicated by a lower induced secretion of IgG and IgA from naïve B cells when compared with HC. In contrast, the amount of IgM produced in cocultures of CD4+CXCR5+ T with autologous naïve B cells of AS/nb patients or HC was comparable. To our knowledge, this is the first functional study directed to assessing the B cell helping capacity of cTfh cells in patients with a rheumatic disease.

Our findings in AS/nb patients are in contrast with the elevated frequency of cTfh and plasmablasts described in autoimmune diseases characterized by autoantibody production [23], [27]–[31]. In fact, it has been proposed that increased numbers of cTfh can be a signature of human immune-mediated diseases [26]. Specifically, a causal relation between accumulation of Tfh cells, autoantibody production and lupus nephritis has been demonstrated in mice [20], and augmented numbers of Tfh counterparts in the peripheral blood of patients with SLE have been associated with disease severity [23]. Conversely, patients with deficiency of CD40-ligand or ICOS demonstrate a severely impaired generation of GC together with decreased circulating CD4+CXCR5+ T cells [40], suggesting that the numbers of cTfh are a reflection of the pool of typical Tfh in lymphoid organs.

The lower frequency of cTfh and plasmablasts in AS/nb might be related with a predominant contribution of innate versus acquired immune mechanisms to the pathogenesis of the disease [3] or with a lower capacity of AS/nb patients to produce T-dependent antibodies. However, AS has not been associated with immunodeficiency, and AS/nb patients are able to mount good antibody responses to vaccination with T-dependent antigens [41]. Alternatively, Tfh could be sequestered in the gut-associated lymphoid tissue (GALT) of AS/nb patients. In fact, subclinical gut inflammation has been described in up to 65% of AS patients [42], and is associated with increased numbers of lymphoid follicles in the ileum and colon [43]; furthermore, increased intestinal permeability has been observed not only in AS patients but also in their relatives [44]. In this context, normal numbers of cTfh cells and plasmablasts in our AS/b patients, the majority of which were receiving infliximab or adalimumab, could parallel an improvement of subclinical gut inflammation induced by these drugs. This would be consistent with studies indicating the leading role of TNF-α in intestinal homeostasis, gut barrier integrity, and pathogenesis of inflammatory bowel disease [45]. However, the levels of serum IgG, IgA and IgM in AS/nb or AS/b patients were discordant with the numbers of cTfh, cTfh subset ratio or plasmablasts: AS/nb patients, with decreased cTfh and plasmablasts, demonstrated normal or high serum IgA concentrations whereas AS/b patients, with normal cTfh and plasmablast numbers, demonstrated increased Ig concentrations. In addition, whereas numbers of cTfh or plasmablasts did not parallel disease activity parameters in AS/nb or AS/b patients, serum IgA in AS/nb or serum IgG, IgA and IgM levels in AS/b patients did, which is consistent with previous observations [36], [37], [39].

Interestingly, patients with AS/b demonstrated increased IgA and IgM concentrations before TNF blockade was started, which did not return to normal levels with treatment, even in patients who achieved a state of inactive or moderate disease activity as determined by ASDAS-CRP values. This suggests that AS/b patients do have a more severe disease as compared with AS/nb subjects, and that increased Ig concentrations in this group are not a side effect of treatment with TNF blockers. In addition, this observation reflects the difficulty in assessing disease activity and severity in AS with the currently available tools [38].

The lack of parallelism of cTfh and plasmablast numbers with serum Ig levels may be explained by two different mechanisms. First, Tfh and plamablasts may be sequestered in the GALT as mentioned above. Also, the altered cTfh subset ratio of AS/nb patients could result from preferential accumulation of Tfh-Th17 over Tfh-Th1 cells in the gut: whereas CCL20, the ligand for CCR6, is normally expressed in the GALT and intestinal epithelium [46], [47], its expression level significantly increases in states of gut inflammation, which conditions local accumulation of CCR6 expressing cells [46], [47]. Local sequestration would lead to decreased cTfh and circulating plasmablast numbers, together with altered cTfh subset ratio, in AS/nb patients. In turn, AS/b patients, with a more severe disease and a more marked alteration of intestinal permeability, would have an even higher accumulation of Tfh and plasmablasts in the GALT, with a higher rate of local Ig production and an increased number of lymphocytes available for recirculation, resulting not only in elevated serum Ig levels [48] but also in apparently normal numbers of cTfh and plasmablasts.

Of note, and as mentioned above, local Tfh sequestration at the inflammatory sites does not seem to occur in autoimmune diseases such as SLE, where increased numbers of total classical Tfh in lymphoid organs are associated with increased numbers of cTfh and autoantibody production [23]. Therefore, local sequestration of Tfh in the gut would be a mechanism operating differentially in AS. An alternative explanation for our findings would be that the number of cTfh does indeed parallel the number of true Tfh in lymphoid organs of AS patients: that is, decreased cTfh in AS/nb or normal cTfh numbers in AS/b patients would be a reflection of a decreased or “normal” pool of classsical Tfh. In this setting, innate immunity with T cell independent class switch recombination, would have a pivotal contribution to Ig production in both AS/nb and AS/b [49]–[51]; in turn, normal numbers of cTfh in AS/b patients would reflect activation of the acquired immune system in patients with a more severe disease [51], [52]. In fact, it has been proposed that the role of innate immunity is predominant at initial stages of bowel inflammation in SpA [51], and as disease progresses or advances in severity, the contribution of acquired immunity tends to become more prominent [51], [52].

Finally, both mechanisms, local Tfh sequestration and increased Ig production by innate mechanisms, may operate simultaneously in AS.

In summary, we have herein described decreased circulating Tfh and plasmablast numbers in AS/nb but not in AS/b patients, in the presence of normal or augmented serum levels of Igs. This observation sheds new light into the pathogenesis of AS and suggests that Tfh cells and plasmablasts may be sequestered in the GALT and/or innate immune mechanisms in the gut have a leading role in the pathogenesis of the disease.
